# Appropriate selection for omalizumab treatment in patients with severe asthma?

**DOI:** 10.1080/20018525.2017.1359477

**Published:** 2017-08-07

**Authors:** Leo Nygaard, Daniel Pilsgaard Henriksen, Hanne Madsen, Jesper Rømhild Davidsen

**Affiliations:** ^a^ Department of Respiratory Medicine, Odense University Hospital, Odense C, Denmark; ^b^ Research Unit of Respiratory Medicine, Clinical Institute, University of Southern Denmark, Odense C, Denmark; ^c^ Department of Clinical Biochemistry and Pharmacology, Odense University Hospital, Odense C, Denmark; ^d^ South Danish Center of Interstitial Lung Diseases (SCILS), Odense University Hospital, Odense C, Denmark

**Keywords:** Compliance, allergy, pharmacotherapy, Asthma Control Test, IgE

## Abstract

**Background**: Omalizumab improves asthma control in patients with uncontrolled severe allergic asthma; however, appropriate patient selection is crucial. Information in this field is sparse.

**Objective**: We aimed to estimate whether potential omalizumab candidates were appropriately selected according to guidelines, and the clinical effect of omalizumab treatment over time.

**Design**: We performed a retrospective observational study on adult patients with asthma treated with omalizumab during 2006–2015 at the Department of Respiratory Medicine at Odense University Hospital (OUH), Denmark. Data were obtained from the Electronic Patient Journal of OUH and Odense Pharmaco-Epidemiological Database. Guideline criteria for omalizumab treatment were used to evaluate the appropriateness of omalizumab candidate selection, and the Asthma Control Test (ACT) to assess the clinical effects of omalizumab at weeks 16 and 52 from treatment initiation.

**Results**: During the observation period, 24 patients received omalizumab, but only 10 patients (42%) fulfilled criteria recommended by international guidelines. The main reasons for not fulfilling the criteria were inadequately reduced lung function, insufficient number of exacerbations, and asthma standard therapy below Global Initiative for Asthma (GINA) step 4–5. Seventeen and 11 patients completed treatment at weeks 16 and 52, with a statistically significant increase in ACT score of 5.1 points [95% confidence interval (CI) 3.1–7.2, *p* = 0.0001] and 7.7 points (95% CI 4.3–11.1, *p* = 0.0005), respectively.

**Conclusion**: Only 42% of the omalizumab-treated patients were appropriately selected according to current guidelines. Still, as omalizumab showed significant improvement in asthma control over time, it is important to keep this drug in mind as an add-on to asthma therapy in well-selected patients.

## Introduction

The main goal of asthma management is to achieve and maintain symptom control and normal activity levels, and to minimise the future risk of exacerbations [1,2]. Inadequately controlled asthma affects both patients and society in terms of days lost from work and school, reduced quality of life (QoL), and avoidable healthcare visits and hospitalisations [3]. Since asthma affects around 300 million people worldwide, this is a major public health problem and managing patients with severe asthma, in particular, can be a challenge [4]. Despite treatment with inhaled corticosteroids (ICS) and long-acting β_2_-agonists (LABA), severe asthma is often uncontrolled and accounts for up to 80% of the total costs of asthma, even though severe asthma represents only 5–10% of the total asthma population [4,5].

Omalizumab is a monoclonal antibody indicated as add-on therapy for patients with severe persistent asthma. Approximately 50% of patients with uncontrolled severe asthma have an immunoglobulin E (IgE)-mediated phenotype [4,6]. IgE plays an important role in allergic asthma and particularly in the acute response to antigens and in the proliferation of airway inflammation [7]. Omalizumab inhibits binding of free IgE to high-affinity receptors on pro-inflammatory cells, which clinically correlates with a reduction in asthma symptoms and exacerbations [8,9].

Clinical trials and observational studies have shown omalizumab to significantly reduce exacerbation rates and frequency of hospitalisations, together with improving both asthma QoL scores and symptom control [4,10–12]. Since uncontrolled severe asthma is a major healthcare problem, it is crucial that potential candidates for omalizumab treatment are selected appropriately, according to current guidelines. Omalizumab is a rather expensive treatment, with a cost per defined daily dose (DDD) of around €50, necessitating good compliance with regular outpatient clinic attendance. Only scant evidence exists on the appropriateness of selection, use, and clinical effectiveness of omalizumab. Hence, the objectives of this study were to assess, first, whether asthma patients treated with omalizumab were selected appropriately according to current guidelines; and secondly, the clinical effect of omalizumab treatment assessed according to changes in asthma symptoms, lung function, asthma control medication, and asthma exacerbations over time.

## Methods

### Design

We performed a retrospective, observational study in adult patients with severe allergic asthma treated with omalizumab in the Region of Southern Denmark during an observational period from 1 October 2006 to 31 March 2015. Data were analysed using longitudinal and individual repetitive cross-sections according to scheduled follow-ups.

### Setting

In the Region of Southern Denmark, examination and treatment of severe asthma, including specialised treatment with omalizumab, are managed at the Department of Respiratory Medicine at Odense University Hospital (OUH). This patient category is primarily assessed in the outpatient clinic, which acts as a secondary and tertiary specialist unit serving a population of nearly one million people aged ≥ 15 years (1 January 2015) [13]. Before receiving omalizumab, all patients go through a preliminary examination about 1 or 2 weeks before the index date, defined as the day of omalizumab initiation, to secure appropriate selection and to determine the correct dose based on weight and IgE level. Omalizumab is indicated only for patients fulfilling the following criteria: age ≥ 6 years; severe persistent asthma; documented positive skin test or *in vitro* reactivity to a perennial aeroallergens; frequent daytime symptoms or night-time awakenings; asthma exacerbations requiring systemic glucocorticoids despite daily high-dose ICS [or leukotriene receptor antagonist (LTRA)] and LABA; weight of 20–150 kg; total IgE at 30–1500 IU/mL; and for patients aged ≥ 12 years, forced expiratory volume in 1 sec (FEV_1_) < 80% [1,14–16]. In accordance with the omalizumab European Union (EU) label and guidelines from the Danish Society of Respiratory Medicine, the treatment effect was evaluated after 16 weeks, and if the treatment was continued further evaluation was performed at annual visits [14,16,17].

### Data sources

The data for this study were obtained from two clinical databases: the Electronic Patient Journal (EPJ) of OUH and the Odense Pharmaco-Epidemiological Database (OPED).

#### EPJ

EPJ contains journal data on patients from all admissions and outpatient clinic visits. The data include medication status, pulmonary function tests (PFTs), Asthma Control Test (ACT) score, and blood sample results. Furthermore, EPJ contains information on hospital electronic medical record systems from all regions, providing an overview of a patient’s medical record in relation to a hospital visit.

Since 2006, the Department of Respiratory Medicine at OUH has systematically registered all patients treated with omalizumab, comprising a total cohort of 32 patients.

#### OPED

Information on reimbursed drug dispensing in the County of Funen has been recorded in the OPED since 1990, and from 1 January 2007 for the entire Region of Southern Denmark (population 1.2 million) [13,18]. Each prescription record includes a person identifier, the date of dispensing, and the brand, active substance, quantity, and form of the drug. The substances and quantities are registered according to the Anatomical Therapeutic Chemical Classification System (ATC) of the World Health Organization and DDD methodology [19]. The indication for treatment and the dosing instruction are not recorded. Drugs not reimbursed and therefore not recorded in the database are over-the-counter drugs and some non-reimbursed prescription drugs, mainly oral contraceptives, hypnotics, sedatives, some antibiotics and intranasal drugs for rhinitis, but also drugs registered only for hospital use, e.g. omalizumab [20].

### Study population

Among the cohort of omalizumab-treated asthma patients, only those from whom we received informed written consent were included. All data were anonymised. Owing to treatment evaluation taking place after 16 weeks, only patients fulfilling 16 weeks of treatment were included for objective 2.

For each included patient, EPJ data related to omalizumab treatment (e.g. PFTs, smoking history, ACT score, and IgE levels) were retrieved and indirectly used for manually validating the asthma diagnosis and the appropriateness of omalizumab treatment. The total number of admissions 1 year before omalizumab initiation and documentation of allergy tests were sought and validated through old records, e.g. former admission journals and discharge summaries.

OPED were used to retrieve data on all redeemed individual medication from 1990 to 31 March 2015. Prescriptions redeemed before omalizumab treatment were used to classify comorbidities (comorbidities according to ATC codes, Appendix I). The date of omalizumab initiation was defined as the index date for the individual patient. As a surrogate marker of compliance with asthma controller medication, i.e. ICS (ATC R03BA), LABA (ATC R03AC12 R03AC13), ICS/LABA (ATC R03AK06, R03AK07, R03AK08, R03AK09, R03AK10, and R03AK11), or LTRA (ATC R03DC), we used a definition of having redeemed prescriptions of either ICS and LABA, or LTRA and LABA 6 months before the individual index date.

### Outcome variables

#### Objective 1

The primary outcome was to determine the proportion of appropriate selected patients initiated with omalizumab on the basis of guideline recommendations [16]. We defined severe persistent asthma according to the European Respiratory Society/American Thoracic Society definition of severe asthma, and the criteria regarding frequent daily or nocturnal symptoms were fulfilled when patients had an ACT score below 20 points, equivalent to uncontrolled asthma [5]. The number of prescriptions for systemic glucocorticoids (ATC H02AB) was used as a surrogate marker of asthma exacerbations before and during treatment with omalizumab. Primary compliance to medication (i.e. at least one redeemed prescription for asthma control medication) was measured using the OPED.

#### Objective 2

**Figure 1.**
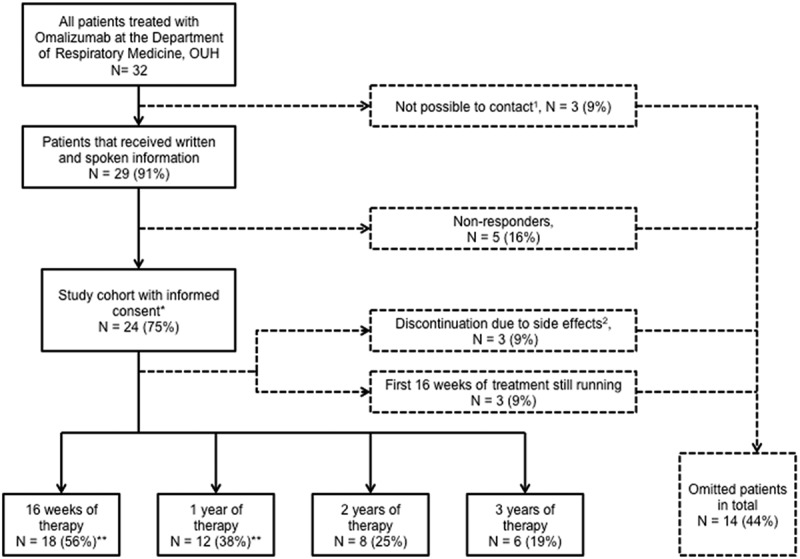
Flowchart for enrolment of patients in the study. The numbers of patients completing 2 and 3 years of therapy were considered too small for analysis. *Patients used for objective 1; **patients used for objective 2. ^1^Cause of missing contact: death (1), missing contact information (2); ^2^side effects registered: general physical discomfort (1), arthralgia (1), headache, fatigue and dyspnoea (1).

Patients completing 16 weeks of omalizumab treatment were included (Figure 1). The primary outcome was asthma control measured by the use of ACT, a simple quantitative tool for assessing asthma control consisting of a validated five-item questionnaire. Each item is scored on a five-point Likert scale from 1 to 5 (1 = worst; 5 = best), and total scores of 5–19 and 20–25 points reflect uncontrolled and well-controlled asthma, respectively. The minimal clinically important difference (MCID) is 3 ACT points [21]. Asthma control was measured by the ACT at the preliminary examination and at scheduled follow-ups. The latter also applied to secondary outcomes such as changes in PFTs, asthma medication status, and number of exacerbations.

On the basis of redeemed prescriptions, comorbidities were categorised using the same categorisation as validated by Kuo et al. [22]; however, corticosteroids for systemic use (ATC H02A) were removed from the rheumatic category owing to an essential overlap with treatment of asthma exacerbations.

### Data analysis

Using the unique civil registration number (CRN) assigned to every Danish citizen, relevant information was retrieved and linked from the above-mentioned data sources. Outcomes according to the objectives were analysed for each patient according to cross-sectional measurements at the index date, at 16 weeks from treatment initiation, and at 52 weeks of follow-up.

We used a paired *t*-test to analyse whether the effects on ACT score and PFT measurements were statistically significant. A Q-Q plot for normal distribution was generated before the use of the paired *t*-test. If a normal distribution was not present, we used the Wilcoxon matched-pairs signed-rank test and Wilcoxon rank-sum test for unmatched data.

Missing values related to objective 1 were registered as ‘criterion not fulfilled’, whereas missing values in relation to objective 2 were treated with pairwise deletion. Statistical analyses were performed using Stata version 13.0 (StataCorp, College Station, TX, USA). A *p* value < 0.05 was considered statistically significant.

According to Danish law, no ethical approval is needed for register-based studies. The Danish Data Protection Agency (J no. 2008-58-0035) approved the study. All patients gave written informed consent.

## Results

In total, 32 patients were treated with omalizumab from 1 October 2006 to 31 March 2015. The flowchart for enrolment is presented in Figure 1 and baseline data are presented in Table 1.

**Table 1.** Baseline characteristics.

Characteristics	Study population (*N* = 24)
Age (years)	43 ± 12
Women	13 (54)
IgE level (IU/mL)	235 ± 190
Body mass index (kg/m^2^)	30.8 ± 6.4
Omalizumab dose (mg/4 weeks)	422 ± 225
Correct omalizumab dose	18 (75)
ACT score ^a^	12.4 ± 4.5
FEV_1_% predicted (L)	2.30 (69) ± 0.83 (21)
FVC% predicted (L)	3.22 (82) ± 0.90 (17)
FEV_1_/FVC	71.0 ± 15.1
PEF (L/min)	365 ± 113
Admissions (in year preceding index date), mean	0.71
Exacerbations (in year preceding index date), mean^b^	2.64
Perennial aeroallergen	23 (96)
GINA step	
4	19 (79)
5	5 (21)
Tobacco	
Never-smoker	12 (50)
Ex-smoker	12 (50)
Comorbidities^b^	
Coronary and peripheral vascular diseases (antiplatelets and anticoagulants)	2 (9)
Hypertension	4 (17)
Hyperlipidaemia	1 (4)
Ischaemic heart disease	1 (4)
Congestive heart disease (/hypertension)	3 (13)
Diabetes	1 (4)
Acid peptic disease	11 (48)
Thyroid disorders	1 (4)
Chronic IBD	1 (4)
Pain (inflammation)	16 (70)
Pain	8 (35)
Depression	5 (22)
Psychotic illness	1 (4)
Ischaemic heart disease (/hypertension)	3 (13)
Comorbidity, frequency^b^	
0–2	12 (52)
3–4	7 (30)
5–6	2 (9)
≥7	2 (9)

Continuous data are shown as mean ± SD and categorical data as number (%).

^a ^Two patients had no registered Asthma Control Test (ACT) score at the index date.

^b ^One patient lacked information about redeemed prescriptions, as the Odense Pharmaco-Epidemiological Database (OPED) the Region of Southern Denmark outside Funen until 2007.

IgE, immunoglobulin E; FEV_1_, forced expiratory volume in 1 sec; FVC, forced vital capacity; PEF, peak expiratory flow; GINA, Global Initiative for Asthma; IBD, inflammatory bowel disease.

### Objective 1

In total, 24 omalizumab-treated patients (54% women) who had undergone a preliminary examination were included for objective 1 (Table 2). Five of these patients (21%) had no redeemed prescriptions for asthma control medication and were therefore categorised as non-compliant with asthma control medication, and in nine patients either data on previous treatment were unavailable (*n* = 2) or criteria for FEV_1_, exacerbations, or ACT score were not met. According to guideline recommendations, 10 patients (42%) of the study population fulfilled all criteria for omalizumab treatment. The number of patients fulfilling the criteria remained unchanged when adding primary compliance with control medication (i.e. ICS, ICS/LABA, and LTRA/LABA) [16].

**Table 2.** Fulfilled omalizumab selection criteria.

Criteria	Fulfilled, *N* (%)
Age ≥ 6 years	24 (100)
Severe asthma	24 (100)
Achieved compliance with asthma control medication	19 (79)^a^
ACT < 20	20 (83)^b^
FEV_1_ < 80% (if ≥ 12 years)	17 (71)
Perennial aeroallergen	23 (96)
≥ 1 exacerbation	19 (79)^c^
Weight [20;150] kg	24 (100)
Total IgE [30;1500] IU/ml	24 (100)
All criteria fulfilled	**10 (42)**

^a ^Two patients lacked information about redeemed prescriptions as the Odense Pharmaco-Epidemiological Database (OPED) did not cover the Region of Southern Denmark outside Funen before 2007.

^b ^Two patients had no Asthma Control Test (ACT) score registered at the index date. The missing values were registered as ACT > 20.

^c ^One patient lacked information about redeemed prescriptions as OPED did not cover the Region of Southern Denmark outside Funen before 2007.

FEV_1_, forced expiratory volume in 1 sec; IgE, immunoglobulin E.

### Objective 2

**Figure 2.**
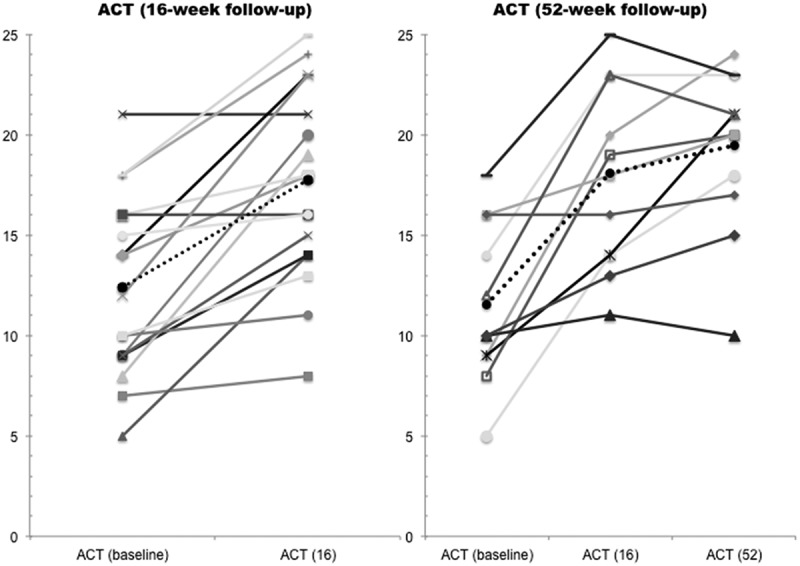
Changes in Asthma Control Test (ACT) score after 16 and 52 weeks of treatment for 17 and 11 patients, respectively. Each line represents a patient and the dotted line represents the mean ACT score. At the 16 and 52 week follow-up the mean ACT score was 17.5 and 19.3, respectively.

During the observation period, 18 and 12 patients completed 16 and 52 weeks of treatment, respectively. However, one patient was excluded owing to a missing ACT score at the index date. A statistically significant increase in mean ACT score by 5.1 points [95% confidence interval (CI) 3.1–7.2, *p* = 0.0001] was found when comparing ACT scores from the index date and at 16 weeks’ follow-up, and 11 patients (65%) had a clinically significant increase of ≥ 3 points in total ACT score. For patients completing 52 weeks of treatment, mean ACT scores increased by 7.7 points (95% CI 4.3–11.1, *p* = 0.0005) and nine (82%) patients had a clinically significant increase in ACT score compared with the index date. Figure 2 illustrates a significant improvement in the proportion of controlled patients, with five (*p* = 0.03) and seven (*p* = 0.01) further patients having ACT scores of ≥ 20 after 16 and 52 weeks of omalizumab treatment, respectively.

Women had a 4.56 point (95% CI 0.84–8.28, *p* = 0.02) higher increase than men in mean ACT score from the index date to the 16 week follow-up, and the same tendency was present after 52 weeks, with a 6.39 point mean ACT score increase (*p* > 0.05) for women. The ACT mean scores increased in the two follow-ups in both genders, but only women had a statistically significant increase.

The mean FEV_1_% predicted for patients completing 52 weeks of omalizumab treatment showed a slight change from the index date to 16 and 52 weeks of follow-up, corresponding to 11 percentage points (95% CI 1–20, *p* = 0.03) and 10 percentage points (95% CI 0–21, *p* = 0.05), respectively. The matching changes in mean FEV_1_ were 0.31 L and 0.29 L, respectively. The remaining PFT measurements showed no significant changes, and are illustrated in Table 3 together with mean biannual exacerbation rates for the patients completing 52 weeks of treatment.

**Table 3.** Development in mean pulmonary function test (PFT) measurements and mean exacerbation rates for patients completing 52 weeks of treatment.

Characteristics	Baseline	16 weeks	52 weeks
FEV_1_% predicted (L)			
(*n* = 12)	2.16 (69) ± 0.67	2.47 (81) ± 0.55	2.45 (81) ± 0.62
FVC% predicted (L)			
(*n* = 12)	3.06 (84) ± 0.75	3.34 (89) ± 0.62	3.27 (89) ± 0.69
PEF (L/min)			
(*n* = 9)^b^	352 ± 56	403 ± 77	402 ± 70
Exacerbations^a^			
(*n* = 11)^b^	1.27 ± 0.9	1 ± 2.1	0.64 ± 1.2

Data are shown as mean (%) ± SD; *n* indicates the number of patients included.

^a ^The exacerbation rates are biannual and account for the mean number of exacerbations (1) during the 180 days preceding the index date, (2) from the index date to 180 days afterwards, and (3) from 180 to 360 days of treatment, respectively.

^b ^Because of no registered peak expiratory flow (PEF) measurements and missing information about redeemed prescriptions from the Odense Pharmaco-Epidemiological Database (OPED), only nine and 11 patients were included in calculations of PEF and exacerbation rate, respectively.

FEV_1_, forced expiratory volume in 1 sec; FVC, forced vital capacity.

The mean number of exacerbations decreased throughout the 52 weeks. However, one patient was responsible for the majority of the total number of exacerbations, corresponding to seven out of 11 (64%) and four out of seven (57%) exacerbations, respectively, after 16 and 52 weeks of omalizumab treatment. Furthermore, we found that the frequency of annual asthma exacerbations reduced by 28% or an average of 0.64 (*p* > 0.05) exacerbations compared to the year before the index date (data not shown).

No significant difference was found in asthma control between appropriately selected versus non-appropriately selected patients when stratifying for variables such as correct omalizumab dose, obesity [body mass index (BMI) > 30 kg/m^2^], smoking status, and compliance with asthma control medication.

## Discussion

The main finding of this study was that only 42% of the patients with severe asthma treated with omalizumab had been appropriately selected according to current guidelines [1,14–16]. Despite this, omalizumab had a significant effect on asthma control, with an increase in mean ACT score of 5.1 and 7.7 at 16 and 52 weeks’ follow-up, respectively, and with the highest mean ACT scores in women and no significant increase in men. Although not statistically significant, a trend towards a reduction in numbers of exacerbations was observed after 52 weeks of omalizumab treatment compared to before initiation of omalizumab treatment. In addition, obesity seemed to be a general trait among all omalizumab-treated patients.

According to objective 1, previous studies have not paid much attention to the appropriateness of selecting patients for omalizumab treatment in clinical settings, which is why there is no direct frame of reference. Changes in the selection criteria during the observation period could, however, have had a negative influence on the overall proportion of well-selected patients, as the criteria used in this study are based on current guideline recommendations [1,14–16]. Thus, comparing the number of treated patients and the possible number of patients eligible for omalizumab treatment seems somewhat contradictory. The current selection seems inadequate owing to a low number of omalizumab-treated patients fulfilling the selection criteria, and more patients are expected to be treated with omalizumab according to asthma prevalence. In Denmark, the prevalence of asthma in adults is around 7%, of whom 8% can be classified as having severe asthma [23,24]. If at least 50% have allergic asthma, 2,800 adults are likely to have severe allergic asthma in the Region of Southern Denmark. This number may be a rough estimate, but the number of treated patients in our study and the expected number of patients treated with omalizumab differ. This difference is probably due to multiple factors, including attitudes towards treatment, symptom neglect among patients, socio-economic factors such as disposable income, the treating physician’s knowledge of severe asthma and available insight into the guidelines, deselection of treatment, and bottlenecks in referral to tertiary asthma centres [25,26]. A major study by Buhl et al. supports this explanation [27].

The results of objective 2 are consistent with findings from previous studies documenting that treatment with omalizumab in patients with severe persistent asthma increases asthma control and reduces exacerbation rates [4]. A large, international, observational study of omalizumab use in 943 patients found similar results according to ACT score, with an increase of 6.1 on average after 12 months of treatment, and mean ACT scores at the index date and after 12 months of 13.0 and 19.1, respectively [12]. However, this study did not aim to assess the appropriateness of selecting candidates for omalizumab. Since the MCID is 3, the increase in ACT score indicates that omalizumab improved asthma control to a level of clinical significance. Because of the small study population, we had very few patients with admissions due to asthma, so it was not possible to perform any significant analysis on the effectiveness of omalizumab in reducing hospitalisations due to asthma.

Women had better asthma control than men at both 16 and 52 weeks. The association between gender and asthma control may be confounded by the higher BMI among women (BMI: women 33 kg/m^2^ and men 28 kg/m^2^; data not shown), as obesity is a known predictor of poor asthma control [2014,^29^]. However, female gender was shown to be associated with worse asthma control in a Spanish cross-sectional study [29].

It is well documented that obesity and uncontrolled asthma are positively associated [30,31]. We found a high mean BMI of nearly 31 kg/m^2^ in our study population, corresponding to obesity, and although our data cannot be used to explore for causality, this could be a potential explanation for a requirement of omalizumab as add-on therapy to achieve asthma control among these patients. However, evidence to support the benefit of omalizumab on asthma control in obese patients is hard to come by, as most studies investigating the effectiveness of omalizumab have only registered weight, not BMI, making consistent comparisons difficult [12,32,33].

### Strengths and limitations

The main strength of the study was the inclusion of all omalizumab-treated patients from a tertiary referral asthma centre covering one million inhabitants aged ≥ 15 years, and not least the manual validation of all data from EPJ, including the asthma diagnosis, sensitisation to perennial aeroallergens, omalizumab treatment, IgE level, BMI, and smoking status. Another strength was the use of electronic pharmacy records, with a high level of completeness, to account for patients’ medication use [34]. As prescription data from OPED were extensive and records completely covered all dispensed drugs, we avoided the problem of recall bias compared with self-reporting, where patients with a chronic disease tend to overestimate their level of compliance [35]. Furthermore, assessing prescription records retrospectively added an additional advantage to our study, since it removed the Hawthorne effect, often seen when patients under observation improve in their adherence to medication. Nonetheless, prescription data account only for primary and not for secondary compliance with medication (i.e. the patient actually takes the medicine) [20]. In this way, valid information on real drug use would have been preferable, e.g. obtained by directly observed therapy or measurement of concentrations of a drug or its metabolite in the blood or urine. However, such approaches have major disadvantages in that they can be rather difficult to administer, expensive, and invasive for the patient.

We may have underestimated the proportion of appropriately selected patients, since missing values in objective 1 were treated as though the criterion was not fulfilled. This leaves a risk of differential misclassification according to OPED data and ACT score. There is also a risk of underestimating exacerbation rates, as patients may have redeemed a large quantity or already have a supply of systemic glucocorticoids at home and could thereby be self-administering when symptoms deteriorate. To obtain a more accurate estimate of exacerbation rates and compliance with asthma control medication, calculation of DDDs could have been an option. The chosen criteria in this retrospective study were based on existing guideline criteria from the European Medicines Agency (EMA), the UK National Institute for Health and Care Excellence (NICE), and the Danish Society of Respiratory Medicine, with only two minor differences. First, in the mentioned guidelines an ACT score < 20 was not an inclusion criterion for omalizumab treatment [14–16]. In our cohort, this criterion excluded four patients (20 patients included versus 24 with informed consent), and if this criterion was omitted three out of the four excluded patients would have fulfilled all the other inclusion criteria, leaving the total number of patients fulfilling all criteria at 13 (54.1%). The second difference was the exacerbation rate. This study included patients with one or more annual exacerbations, whereas NICE and EMA refer to ‘multiple exacerbations’, meaning more than one [14,15]. If the ‘multiple exacerbation’ criterion was used in our cohort and defined as more than two annual exacerbations, this would exclude five of the 19 patients registered with exacerbations, leaving the total number of patients fulfilling all criteria at five (20.8%). Therefore, the applied and potentially restrictive ACT criterion in our cohort was very likely to have been be counterbalanced by the applied exacerbation criterion. Hence, we do not think that patients were excluded from omalizumab treatment differently from other asthma centres using the above-mentioned guideline criteria, and as such we find that the selected patients reliably represent omalizumab candidates outside our region and country.

Another limitation of our study was the lack of power owing to the small study population. However, since we included all patients treated with omalizumab in the Region of Southern Denmark, the only option to enhance power would be to conduct a multicentre study. We had an acceptable inclusion response rate of 75%, but it still entails a risk of selection bias. As the study was retrospective and did not require intervention, responders and non-responders to omalizumab treatment should have had equal incentive to provide informed consent. However, responders might have been more enthusiastic about participating (responders *n* = 24 versus non-responders *n* = 5) (Figure 1), which could have caused a risk of healthy volunteer bias, a subtype of selection bias.

## Conclusion

This study found that only 42% of the patients with severe asthma treated with omalizumab were appropriately selected according to current guidelines. Yet, omalizumab is an important add-on treatment option for well-selected asthma patients, as demonstrated by a significant improvement in asthma control. Future studies should focus on how to improve the selection of patients for omalizumab treatment and encourage identification of specific determinants of patients who are likely to benefit from the treatment, rather than the use of predetermined baseline characteristics.
